# Refractory Pembrolizumab-Induced Myocarditis-Myositis-Myasthenia Gravis Overlap Syndrome With Concurrent Hepatitis and Thyroiditis in Endometrial Carcinoma: A Case Report

**DOI:** 10.7759/cureus.110341

**Published:** 2026-06-06

**Authors:** Deepika Beereddy, Durga Naga Malleswara Rao Jonnalagadda, David R Murillo-Garcia

**Affiliations:** 1 Department of Internal Medicine, Reading Hospital-Tower Health, West Reading, USA; 2 Department of Cardiology, Reading Hospital-Tower Health, West Reading, USA

**Keywords:** abatacept, endometrial carcinoma, immune checkpoint inhibitor-associated myocarditis, immune-checkpoint inhibitor (ici), immune-related adverse events (iraes), myocarditis-myositis-myasthenia gravis overlap syndrome, ruxolitinib, seronegative myasthenia gravis, serous endometrial carcinoma, single-dose pembrolizumab

## Abstract

Immune checkpoint inhibitors (ICIs) have transformed oncologic care but can trigger a spectrum of immune-related adverse events. The triad of myocarditis, myositis, and myasthenia gravis, termed MMM overlap syndrome, is a rare but life-threatening complication. Reports have predominantly involved melanoma and lung cancer; cases of endometrial carcinoma have rarely been described.

A 77-year-old woman with stage IV serous endometrial carcinoma presented 24 days after a single dose of carboplatin, paclitaxel, and pembrolizumab, with progressive weakness, ptosis, diplopia, and respiratory compromise. Initial evaluation revealed creatine kinase (CK) of 13,000 IU/L, peak high-sensitivity troponin I of 15,000 ng/L, a new right bundle branch block (RBBB) with intermittent high-degree atrioventricular (AV) block, transaminitis, and thyrotoxicosis, indicating concurrent four-organ toxicity. Acetylcholine receptor (AChR), MuSK, and LRP4 antibodies were all negative, consistent with seronegative ICI-induced myasthenia gravis. She was treated with pulse corticosteroids and plasma exchange (PLEX), with rituximab added after the second session for an inadequate response. Persistent myocarditis with sustained troponin elevation prompted further escalation to abatacept and ruxolitinib. Symptoms and serum biomarkers subsequently improved, and she was discharged on continued corticosteroids and ruxolitinib with a planned graded taper.

To our knowledge, this is the second reported case of MMM overlap syndrome in endometrial carcinoma and the first implicating pembrolizumab in this malignancy. The case is distinguished by refractory disease requiring four sequential lines of immunosuppression, concurrent immune-mediated hepatitis and thyroiditis, and a seronegative myasthenia gravis profile, consistent with the more heterogeneous, non-antibody-mediated mechanisms increasingly recognized in ICI-induced disease. It informs treatment escalation in refractory cases and underscores three principles essential to managing this high-mortality syndrome: early recognition, systematic screening for all syndromic components once any one is identified, and timely escalation through multiple lines of immunosuppression when disease proves refractory.

## Introduction

Immune checkpoint inhibitors (ICIs) have transformed the treatment landscape for a wide range of malignancies. By blocking inhibitory receptors such as programmed cell death protein-1 (PD-1), its ligand (PD-L1), and cytotoxic T-lymphocyte-associated antigen-4 (CTLA-4), these agents restore T-cell-mediated antitumor immunity and have become integral components of therapeutic regimens for over 20 different cancer types [1,2]. However, the same mechanism that drives their therapeutic efficacy (augmented T-cell activation and loss of self-tolerance) can result in immune-related adverse events (irAEs), affecting virtually any organ system [3].

ICI-associated myocarditis may co-occur with myositis and myasthenia gravis, constituting the myocarditis, myositis, and myasthenia gravis (MMM) overlap syndrome. It is a rare complication of ICI therapy (<1%) and typically presents after a median of 3-4 weeks following ICI initiation [4,5]. MMM overlap syndrome is highly fatal, with in-hospital mortality approaching 38% [4,6]. Melanoma and lung cancer are the two most common malignancies associated with this syndrome, followed by renal cell carcinoma and thymoma [4]. To our knowledge, only one prior case of MMM overlap syndrome has been reported in endometrial carcinoma, following dostarlimab therapy [7].

Here, we present a case of pembrolizumab-induced MMM overlap syndrome with concurrent hepatitis and thyroiditis after a single cycle of chemoimmunotherapy for stage IV endometrial carcinoma. The patient developed intermittent high-degree atrioventricular (AV) block and progressive respiratory compromise and required escalation through four lines of immunosuppressive therapy, including high-dose corticosteroids, plasma exchange (PLEX), rituximab, and combination abatacept with ruxolitinib, ultimately achieving clinical improvement. This case illustrates the importance of early multisystem toxicity recognition, the potential for severe cardiac conduction disease as a manifestation of ICI myocarditis, and the real-world implementation of emerging targeted immunosuppressive strategies, including combination abatacept and ruxolitinib, in a critically ill patient.

## Case presentation

A 77-year-old woman with a past medical history of hypertension, hyperlipidemia, and stage IV serous endometrial carcinoma, status post total abdominal hysterectomy with bilateral salpingo-oophorectomy approximately two months prior, presented from the oncology infusion center with generalized weakness and persistent fatigue. She had completed cycle 1 of chemoimmunotherapy 24 days earlier, consisting of carboplatin, paclitaxel, and pembrolizumab. She also reported new right eyelid drooping beginning one day prior to presentation. She denied headache, focal weakness, speech difficulty, or other neurologic complaints. She denied chest pain, palpitations, abdominal pain, bloating, vaginal bleeding, nausea, or vomiting. Appetite was reported as fair. She had no known drug allergies and denied tobacco use, illicit substance use, and significant alcohol consumption. Home medications included amlodipine 2.5 mg PO daily, lisinopril 40 mg PO daily, atorvastatin 10 mg PO nightly, and cetirizine 10 mg PO daily.

**Figure 1:**
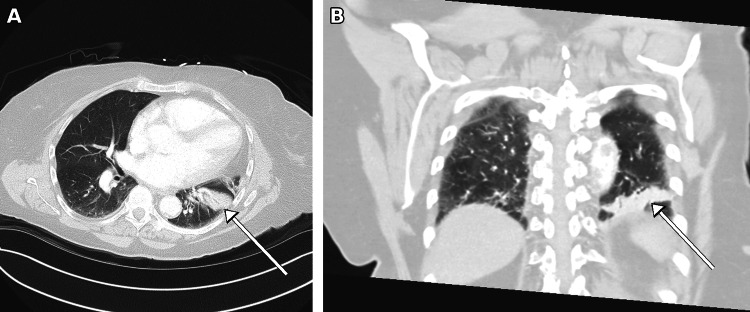
Contrast-enhanced computed tomography angiogram of the chest. (A) Axial image demonstrating patchy consolidation in the left lower lobe (arrow). (B) Coronal reformat confirming the basal location of the left lower lobe consolidation (arrow).

On presentation, the patient was tachycardic and tachypneic, and a sepsis protocol was initiated. Laboratory evaluation revealed leukocytosis (white blood cell count 15.0 × 10⁹/L, absolute neutrophil count 10.86 × 10⁹/L), markedly elevated transaminases (aspartate aminotransferase (AST) 1,242 IU/L, reference <34 IU/L; alanine aminotransferase (ALT) 758 IU/L, reference 10-49 IU/L), and mild acute kidney injury (creatinine 1.19 mg/dL, estimated glomerular filtration rate approximately 47 mL/min/1.73 m²). Hemoglobin and platelet counts were stable. Computed tomography angiography (CTA) of the chest demonstrated left lower lobe consolidation concerning for pneumonia, without evidence of pulmonary embolism (Figure 1). CT of the abdomen and pelvis showed no acute intra-abdominal pathology, and the liver appeared morphologically normal. Intravenous fluids and broad-spectrum antibiotics (piperacillin-tazobactam) were initiated after blood cultures were obtained. Blood cultures subsequently returned negative, and antibiotics were discontinued on hospital day 3, given the absence of respiratory symptoms and low overall clinical suspicion for pneumonia; she was monitored off antibiotics without clinical deterioration.

Thyroid studies revealed a suppressed thyroid-stimulating hormone (TSH) of 0.113 µIU/mL (reference 0.55-4.78 µIU/mL) and elevated free T4 of 2.11 ng/dL (reference 0.89-1.76 ng/dL), consistent with thyrotoxicosis. TSH receptor and thyroid peroxidase antibodies were negative. Coupled with palpitations, night sweats, and 10 pounds of unintentional weight loss since starting chemotherapy, the picture was most consistent with ICI-induced thyroiditis. Given its typical self-limited thyrotoxic phase preceding hypothyroidism, treatment was deferred in the absence of severe symptoms, with serial thyroid function monitoring planned.

Further laboratory evaluation revealed a markedly elevated creatine kinase (CK) of 13,000 IU/L (reference 34-146 IU/L) and a high-sensitivity troponin I (hs-TnI) that peaked at 15,000 ng/L (reference ≤34 ng/L) on hospital day 2 and decreased to 8,094 ng/L on hospital day 3. Electrocardiography demonstrated a new right bundle branch block (RBBB). Transthoracic echocardiography (TTE) was ordered, and a myositis panel was sent; the panel subsequently returned negative on hospital day 6. Given the constellation of findings (elevated transaminases, markedly elevated CK, and troponin, new conduction abnormalities, and right eyelid ptosis), the clinical presentation was consistent with pembrolizumab-induced MMM overlap syndrome (myocarditis, myositis, and myasthenia gravis) with concurrent immune-mediated hepatitis and thyroiditis.

Neurology was consulted for evaluation of the right eyelid ptosis. On further history, the patient reported that her vision had initially felt unusual, with visual spots followed by persistent diplopia that was worse on rightward gaze. The diplopia had resolved after a few days, but she subsequently developed right eyelid drooping one day prior to admission. She had recently been evaluated by ophthalmology for right eye macular degeneration. Neurologic examination was notable for complete ptosis and bilateral ophthalmoplegia. A single-breath count of 23 and a vital capacity of 824 mL were documented, concerning for neuromuscular respiratory involvement. CTA of the chest was negative for thymoma. Based on the severity of the presentation, neurology recommended initiation of intravenous methylprednisolone 1 g daily and therapeutic PLEX for five sessions. Negative inspiratory force (NIF) and vital capacity were monitored every six hours. After three days of pulse-dose methylprednisolone (1 g daily), the dose was transitioned to 1 mg/kg/day. Electromyography was deferred, as results would not alter immediate management, given the high clinical suspicion and ongoing treatment with corticosteroids and PLEX. Pentamidine was administered for *Pneumocystis jirovecii* pneumonia (PJP) prophylaxis, given the patient's inability to tolerate trimethoprim-sulfamethoxazole due to oropharyngeal dysphagia.

**Figure 2:**
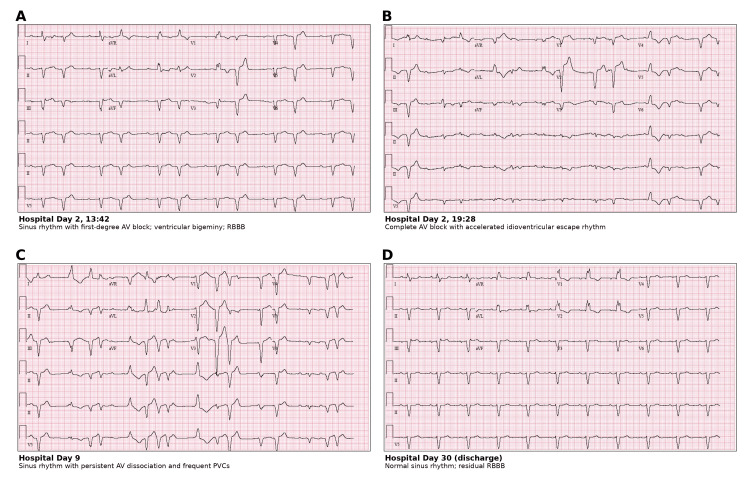
Serial 12-lead electrocardiograms documenting the evolution and resolution of immune checkpoint inhibitor-associated atrioventricular block. (A) Hospital day 2 (13:42): sinus rhythm with first-degree atrioventricular block, frequent premature ventricular complexes in a bigeminal pattern, and right bundle branch block. (B) Hospital day 2 (19:28): complete atrioventricular block with accelerated idioventricular escape rhythm. (C) Hospital day 9: sinus rhythm with persistent atrioventricular dissociation and frequent premature ventricular complexes, indicating ongoing conduction-system inflammation. (D) Hospital day 30 (prior to discharge): restoration of normal sinus rhythm with intact atrioventricular conduction; right bundle branch block persists as residual conduction-system injury. AV: atrioventricular, PVC: premature ventricular complex, RBBB: right bundle branch block.

**Figure 3:**
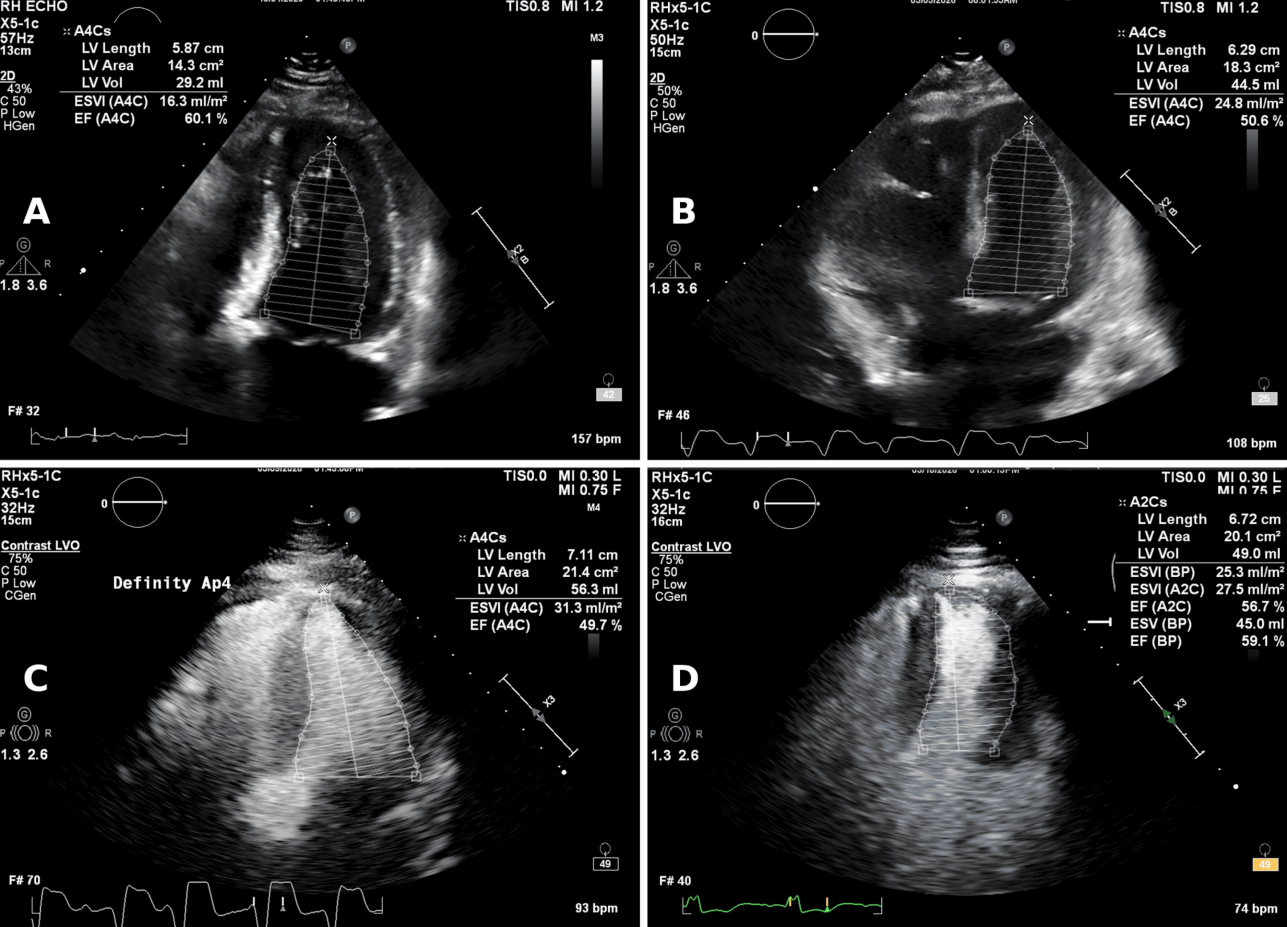
Serial transthoracic echocardiograms demonstrating left ventricular ejection fraction during pembrolizumab-induced MMM overlap syndrome. (A) Baseline echocardiogram (four months prior) showing normal systolic function, with an LVEF of 60%. (B) Echocardiogram on day 3 demonstrating a decline in systolic function to LVEF 48%. (C) Definity contrast-enhanced study on day 7 confirming continued mild systolic dysfunction, with an LVEF of 45%. (D) Follow-up Definity contrast-enhanced echocardiogram on day 16 demonstrating recovery of systolic function, with an LVEF of 59% after immunosuppressive therapy.

Cardiology evaluation revealed a conduction abnormality with frequent premature ventricular complexes (PVCs), RBBB, and intermittent high-degree AV block. During the initial evaluation, the patient maintained sinus rhythm with PVCs in a bigeminal pattern. On the same day, she developed intermittent complete AV block with a ventricular escape rhythm and occasional capture beats and fusion complexes, alternating with ventricular bigeminy. Prophylactic temporary pacing was deferred as she remained hemodynamically stable, and her rhythm gradually improved over time (Figure 2). TTE on hospital day 3 demonstrated new left ventricular systolic dysfunction with a left ventricular ejection fraction (LVEF) of 48%, a decline from her baseline LVEF of 60% four months earlier (Figure 3).

**Figure 4:**
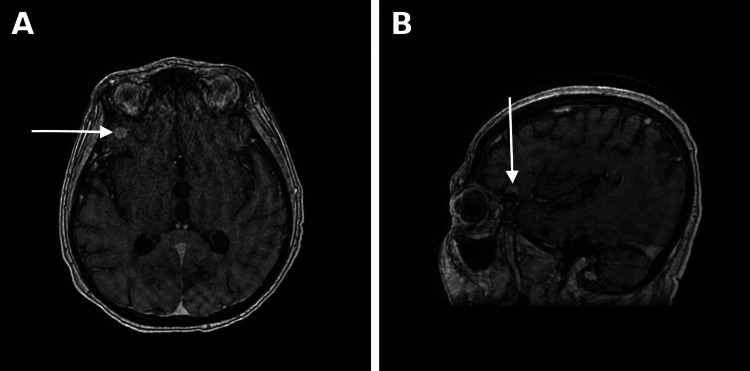
MRI of the brain with and without IV contrast demonstrating a 7 mm extra-axial enhancing mass with a broad dural base along the right greater wing of the sphenoid (arrows), with imaging features suggestive of meningioma versus dural metastasis. (A) Axial view. (B) Sagittal view.

PLEX was initially performed on an alternate-day schedule (days 2, 4, and 6). Given persistent myasthenia gravis symptoms despite the first two sessions of PLEX and pulse-dose corticosteroids, rituximab (500 mg/m², extrapolated from B-cell depletion protocols in the absence of standardized dosing for ICI-associated myasthenia gravis) was administered on hospital day 5. Because of ongoing respiratory involvement and ophthalmoplegia, and in anticipation of escalation to abatacept, the PLEX schedule was subsequently intensified to daily exchange (days 7 and 8) to shorten the overall course and minimize the delay before abatacept administration, since concurrent plasmapheresis would be expected to remove the therapeutic antibody. An MRI of the brain (Figure 4) performed during this period incidentally identified a 7 mm mass at the right greater wing of the sphenoid, with a differential of meningioma versus dural metastasis; short-interval outpatient follow-up imaging was recommended.

By hospital day 8, troponin I remained significantly elevated at 3,140 ng/L. The patient continued to have bilateral ophthalmoplegia and significant ptosis, with a poor vital capacity (640 mL) despite a normal NIF (−60 cmH₂O). She had developed a new oxygen requirement on hospital day 6, requiring up to 4 L/min via nasal cannula, which was gradually weaned to 1-2 L/min by the time of discharge. Liver function tests and CK had improved substantially. Acetylcholine receptor (AChR) binding, muscle-specific kinase (MuSK), and lipoprotein receptor-related protein 4 (LRP4) antibodies, drawn on hospital day 2, all returned negative on hospital day 24, consistent with seronegative ICI-associated myasthenia gravis. A repeat TTE on hospital day 7 demonstrated an LVEF of 45%, elevated left atrial pressure, dilated right ventricle with reduced right ventricular function, and a dilated left atrium, without wall motion abnormalities.

**Figure 5:**
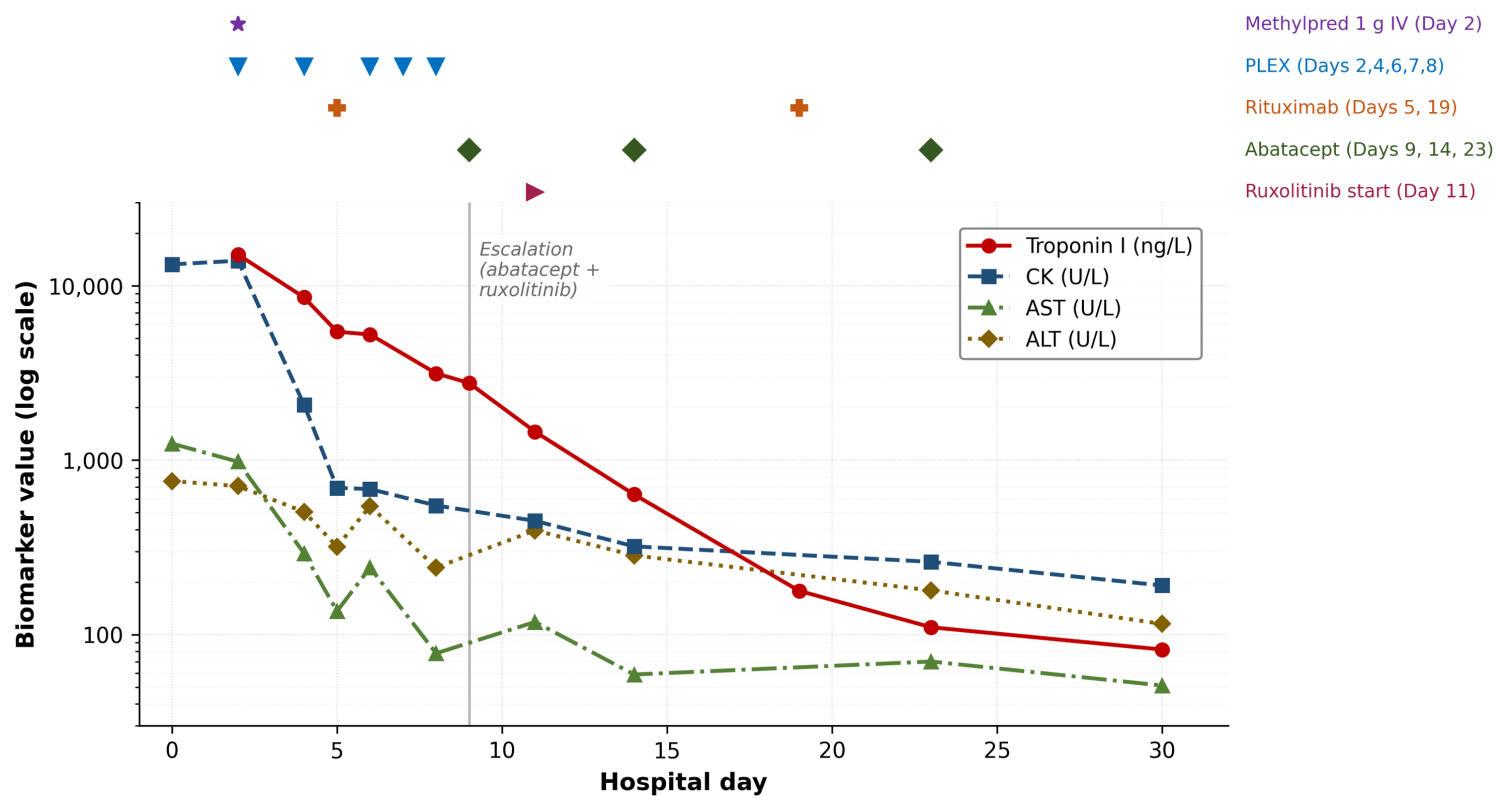
Serial trends in cardiac and muscle/liver enzyme biomarkers across the hospital course. Troponin I, creatine kinase (CK), aspartate aminotransferase (AST), and alanine aminotransferase (ALT) are plotted on a logarithmic Y-axis against hospital day. Symbols above the plot mark therapeutic interventions: pulse-dose methylprednisolone 1 g IV (day 2), therapeutic plasma exchange (PLEX; days 2, 4, 6, 7, and 8), rituximab 500 mg/m² (days 5 and 19), abatacept 20 mg/kg (days 9, 14, and 23), and ruxolitinib 15 mg orally twice daily (day 11). The vertical reference line marks treatment escalation to combination abatacept and ruxolitinib for refractory disease, after which all four biomarkers demonstrated sustained downtrend.

Given persistently elevated troponin I and respiratory weakness despite intravenous methylprednisolone and five sessions of PLEX, treatment was escalated on hospital day 9. Based on emerging evidence supporting abatacept and ruxolitinib in fulminant ICI-myocarditis, abatacept was initiated at a dose of 20 mg/kg, with a planned schedule of three doses administered on days 1, 5, and 14 of the abatacept regimen (hospital days 9, 14, and 23). Ruxolitinib was started on hospital day 11 at 15 mg orally twice daily and was continued at the same dose at the time of discharge, with a planned outpatient taper as detailed below. A second dose of rituximab (500 mg/m²) was administered on hospital day 19 (two weeks after the first dose), and the patient completed all three planned doses of abatacept. Laboratory studies demonstrated a continued downward trend in CK, troponin I, and AST/ALT, with serial biomarker trajectories shown in Figure 5. Corticosteroids were tapered from 80 mg/day to 60 mg/day during hospitalization, with troponin levels monitored with a goal of de-escalation to CTCAE grade 1-2 cardiotoxicity [8]. The complete chronology of clinical events, biomarker trends, and therapeutic interventions is summarized in Table 1.

**Table 1:** Clinical timeline of pembrolizumab-induced MMM overlap syndrome Day 0 = day of hospital presentation. Pembrolizumab + carboplatin + paclitaxel was administered approximately 24 days prior to presentation. AChR: acetylcholine receptor, AV: atrioventricular, CK: creatine kinase, CPK: creatine phosphokinase, ECG: electrocardiogram, EF: ejection fraction, EMG: electromyography, ICI: immune checkpoint inhibitor, ICU: intensive care unit, LA: left atrium, LFT: liver function test, LLL: left lower lobe, LRP4: low-density lipoprotein receptor-related protein 4, MG: myasthenia gravis, MMM: myocarditis, myositis, myasthenia gravis, MuSK: muscle-specific kinase, NIF: negative inspiratory force, PE: pulmonary embolism, PJP: *Pneumocystis jirovecii* pneumonia, PLEX: plasma exchange, PVC: premature ventricular complex, RBBB: right bundle branch block, RV: right ventricle, SBC: single breath count, SIRS: systemic inflammatory response syndrome, TPMT: thiopurine methyltransferase, TPO: thyroid peroxidase, TSH: thyroid-stimulating hormone, TSH-R: thyroid-stimulating hormone receptor, TTE: transthoracic echocardiogram, VC: vital capacity, WMA: wall motion abnormality.

Hospital day	Clinical events	Key diagnostics	Treatments/interventions
-	Cycle 1 of chemoimmunotherapy (~24 days before presentation)	-	Carboplatin + paclitaxel + pembrolizumab
Day 0	Presented from oncology infusion center: generalized weakness, persistent fatigue since starting treatment, new right eyelid ptosis (1 day prior), double vision, worse on right gaze, palpitations, night sweats, 10-pound weight loss, tachycardic, tachypneic; met SIRS criteria	WBC, 15.0 × 10⁹/L; ANC, 10.86 × 10⁹/L; AST, 1,242 IU/L; ALT, 758 IU/L; Cr, 1.19 mg/dL (eGFR ~47 mL/min/1.73 m²); TSH, 0.113 µIU/mL; free T4 2.11 ng/dL; CK, 13,000 IU/L; new RBBB on ECG CTA chest; LLL consolidation; no PE; no thymoma	IV fluids (1 L), piperacillin-tazobactam (sepsis protocol), TSH-R Ab, TPO Ab, myositis panel sent, echo ordered
Days 1-2	Diagnosed with ICI-induced myositis, myasthenia, myocarditis, hepatitis, and thyroiditis; complete ptosis, bilateral ophthalmoplegia SBC 23, VC 824 mL; transferred to ICU. Heme-Onc clinical impression: MMM overlap syndrome with concurrent immune-mediated hepatitis and thyroiditis	Troponin I, 15,000 ng/L; NIF/VC monitoring q6h initiated	Methylprednisolone 1 g/day IV × 3 days, plasma exchange (PLEX) #1 on day 2
Days 3-4	Intermittent complete AV block with ventricular escape, alternating with ventricular bigeminy; hemodynamically stable (prophylactic pacing not pursued); frequent PVCs, RBBB on telemetry	TTE on day 3: EF 48% (decline from a baseline EF of 60% ~4 months ago); brain MRI: 7 mm mass (meningioma vs. metastasis)	Continued steroids + PLEX #2 on day 4
Day 5	Persistent MG symptoms after 2 PLEX sessions; transient tachycardia, tachypnea, feeling hot during rituximab infusion (completed at slower rate)	Rhythm improving; frequent ventricular ectopy persists	Rituximab 500 mg/m² (first dose), methylprednisolone transitioned to 1 mg/kg/day
Days 6-8	Continued ophthalmoplegia and ptosis, poor VC (640 mL), normal NIF (-60), persistent respiratory muscle weakness; new O2 requirement (4 L/min nasal cannula); LFTs and CK improving	TPO Ab: negative; AChR-binding Ab: negative; MuSK and LRP4 Ab pending; TPMT level sent (for steroid-sparing planning); EMG deferred; TTE day 7: EF 45%, dilated LA, elevated LA pressure, dilated RV with reduced RV function, no WMA, troponin I 3,140 ng/L (persistently elevated)	Pentamidine for PJP prophylaxis, continued methylprednisolone at 1 mg/kg/day PLEX #3, #4, #5 on days 6, 7, and 8
Day 9	Treatment escalated due to persistent troponin elevation and respiratory weakness despite steroids + 5 PLEX	CPK, troponin I, AST/ALT trending down; ECG: frequent PVCs, RBBB, intermittent advanced AV block	Abatacept 20 mg/kg, first dose on day 9; ruxolitinib 15 mg PO BID, started on day 11 (planned duration: ~2 months)
Day 14	Clinical improvement continuing	Continued down-trend of troponin, CPK, AST/ALT	Abatacept 20 mg/kg, second dose
Day 19	Ongoing clinical recovery	Serial labs improving	Rituximab 500 mg/m², second dose
Day 23	Significant improvement in ptosis, diplopia, dysphagia, respiratory effort	Troponin reached grade 1-2 cardiotoxicity	Abatacept 20 mg/kg, third (final) dose
Day 30 Discharge	Symptoms of ptosis, diplopia, dysphagia, and generalized weakness significantly improved; persistent wide QRS/RBBB; pembrolizumab permanently discontinued	Serial labs trending toward baseline. Repeat echocardiogram on day 16 showed improved EF of 59%	Prednisone 40 mg daily at discharge (taper 10 mg weekly); planned outpatient cardiac MRI follow-up: Gyn-Onc, Med-Onc, cardiology, neurology

At the time of discharge on hospital day 30, the patient had sustained sinus rhythm with persistent RBBB but no further high-degree AV block, with a troponin I of 82 ng/L. The most recent echocardiogram, performed 14 days prior to discharge (hospital day 16), demonstrated recovery of left ventricular function with an LVEF of 59%. Her symptoms of right eyelid ptosis, diplopia, dysphagia, and generalized weakness had improved significantly over the hospital course. She was discharged on prednisone 40 mg daily with a planned weekly 10 mg taper, along with serial monitoring of AST, ALT, CK, and troponin. The decision was made to permanently discontinue pembrolizumab.

Post-discharge follow-up demonstrated continued clinical improvement. During inpatient rehabilitation, she developed hypothyroidism as the expected sequela of her earlier ICI-induced thyroiditis and was started on levothyroxine 25 mcg daily, with planned repeat thyroid function testing in three months. At the hematology clinic follow-up on post-discharge day 14, she remained on prednisone 30 mg daily with a planned weekly taper of 10 mg, and continued ruxolitinib 15 mg orally twice daily with plans to maintain this dose for approximately two months before initiating a gradual taper to discontinuation; abrupt withdrawal was deliberately avoided given the risk of immune rebound. Serial laboratory monitoring of troponin I, CK, AST, and ALT was scheduled every two weeks. PJP prophylaxis was transitioned from inpatient pentamidine to oral trimethoprim-sulfamethoxazole following resolution of oropharyngeal dysphagia, and gastrointestinal prophylaxis with lansoprazole was continued. At the time of discharge from inpatient rehabilitation 19 days after hospital discharge, laboratory markers had substantially improved: troponin I 39 ng/L, CK 100 IU/L, AST 23 IU/L, and ALT 72 IU/L. Outpatient follow-up was arranged with cardiology (including consideration of cardiac MRI), neurology, and gynecologic oncology for ongoing management of her endometrial carcinoma. At four weeks post-discharge, she remained clinically stable, with no rehospitalization or recurrence of myasthenic, cardiac, or hepatic symptoms, and continued to attend scheduled outpatient appointments.

## Discussion

This is a case of pembrolizumab-induced MMM overlap syndrome presenting approximately four weeks after therapy in a 77-year-old woman with stage IV endometrial carcinoma. The most common presenting symptoms of MMM overlap syndrome are muscle weakness, shortness of breath, ptosis, and diplopia, all of which were observed in our patient [4]. Other irAEs, including thyroiditis and hepatitis, may co-occur [3]. Diagnostically, a significant elevation of cardiac biomarkers (troponin and CK) is associated with a high likelihood of myocarditis once myocardial infarction has been excluded [4,6], and the presence of concurrent arrhythmias further strengthens the diagnosis [5]. Although cardiac MRI and endomyocardial biopsy are the definitive diagnostic modalities for ICI-induced myocarditis, the high fatality of the condition warrants immediate treatment without delay [6,9]. Our patient had a significant elevation of troponin along with conduction abnormalities on ECG, including intermittent high-degree AV block. Continuous telemetry monitoring was maintained until conduction normalized. A persistent RBBB remained at discharge, consistent with residual conduction-system injury.

The most common causes of mortality in MMM overlap syndrome are respiratory failure [6,9] and cardiac arrhythmias [10], making close monitoring for worsening myasthenic symptoms and respiratory decline essential in these cases [9,11,12]. Various conduction abnormalities, including AV block, have been reported in ICI-induced myocarditis, and fatal ventricular arrhythmias have also been described [5,10]; some reported cases have required pacemaker placement in addition to immunosuppressive therapy [4,7].

Myositis is reported in 25%-40% of patients with ICI-associated myocarditis, and concurrent myasthenia gravis co-occurs in approximately 10%-11% of cases [4,13]; together, these entities constitute the MMM overlap syndrome [4]. The recommended diagnostic modalities for ICI-related myositis include CK elevation, consideration of MRI imaging, and muscle biopsy for definitive diagnosis [6]. In our patient, the combination of elevated CK in the setting of recent ICI therapy, alongside concurrent irAEs, supported the clinical diagnosis of both myositis and myasthenia gravis. Our patient had classic symptoms of ptosis, diplopia, weakness of facial muscles, and dysphagia suggestive of myasthenia gravis [4]. As AChR, MuSK, and LRP4 antibody results were not immediately available, the diagnosis was made clinically; all three antibodies subsequently returned negative, consistent with seronegative ICI-associated myasthenia gravis.

The major pathogenesis is considered to be the activation and infiltration of CD8+ T cells into the myocardium and skeletal muscles, based on findings from endomyocardial and muscle biopsies that demonstrate T-cell infiltration [14]. Another proposed mechanism is that shared antigens, most notably α-myosin, between myocardium and skeletal muscle may explain the co-occurrence of these conditions as an overlap syndrome [15]. In contrast, the pathogenesis of ICI-induced myasthenia gravis is less clearly established, with some cases showing anti-AChR antibodies supporting a B-cell-mediated humoral mechanism, while seronegative cases suggest a less well-characterized, heterogeneous mechanism [16]. These mechanistic insights inform current treatment strategies, including the use of CTLA-4 agonists (abatacept) and JAK inhibitors (ruxolitinib) to reverse T-cell-mediated injury [9].

ICI-associated myasthenia gravis frequently occurs without detectable autoantibodies [16], and antibody negativity should not exclude the diagnosis when characteristic features such as ptosis, ophthalmoplegia, dysphagia, and respiratory muscle weakness develop after ICI exposure. The triple-seronegative profile in our patient is consistent with a predominantly T-cell-mediated immune process and supports the use of T-cell-targeted therapies such as abatacept and ruxolitinib in refractory ICI-associated MMM overlap syndrome.

Co-occurrence of thyroiditis did not require additional treatment, as ICI-associated thyroiditis is typically self-limiting and transitions to hypothyroidism, requiring only serial monitoring of thyroid function to ensure resolution of the hyperthyroid phase [3,6]. Our patient ultimately developed hypothyroidism during the recovery period and was started on levothyroxine, consistent with this expected natural history. Similarly, the patient's transaminase elevation attributable to ICI-related hepatitis was managed with close monitoring and resolved with the ongoing immunosuppression, not requiring organ-specific escalation.

The first-line treatment for ICI-associated myocarditis and myositis is high-dose corticosteroids [6]. Our patient was accordingly started on pulse-dose methylprednisolone at 1 g/day for three days. However, high-dose corticosteroids carry a risk of worsening myasthenia gravis [4,6]. Given this risk and the high fatality of MMM overlap syndrome [4,13], our patient was started on therapeutic PLEX early [6]. Rituximab has been used in patients refractory to initial treatment [6,7], and our patient was started on rituximab after the second session of PLEX.

Emerging research has shown that the addition of abatacept (a CTLA-4 fusion protein) and ruxolitinib (a JAK inhibitor) substantially improves mortality in severe ICI-associated myocarditis, particularly when complicated by respiratory muscle weakness [9]. This combination was initiated in our patient because of severe myocarditis and evidence of respiratory muscle weakness, indicated by declining vital capacity (VC), negative inspiratory force (NIF), and a new oxygen requirement, despite three prior lines of immunosuppressive therapy. The abatacept dose was guided by the ACHLYS trial protocol [11], and an additional ongoing trial (ATRIUM) is further investigating optimal abatacept dosing, with major adverse cardiac events as the primary outcome [12]. The patient's ptosis, respiratory muscle weakness, oxygen requirement, and dysphagia improved substantially with this four-line regimen: high-dose IV corticosteroids, PLEX, rituximab, and combination abatacept plus ruxolitinib. Troponin was monitored daily until cardiotoxicity improved to grades 1-2 [6].

Our case delineates only the second reported case of ICI-related MMM overlap syndrome in endometrial carcinoma [7]. This case is distinguished by an earlier onset after pembrolizumab initiation (24 days vs. 81 days in the prior dostarlimab case), the development of intermittent high-degree atrioventricular block, a triple-seronegative myasthenia gravis profile, and the need for escalation through four sequential lines of immunosuppression, including abatacept and ruxolitinib. Notably, the conduction abnormality gradually resolved over the hospital course, while persistent troponin elevation and respiratory muscle weakness prompted escalation to combination abatacept and ruxolitinib. The mechanism by which certain malignancies may be predisposed to MMM overlap syndrome remains unclear, and further research, including ongoing clinical trials, is needed to delineate standard treatment approaches for this often fatal condition [12].

This report has several limitations. Cardiac MRI and endomyocardial biopsy, the gold-standard diagnostic modalities for ICI-associated myocarditis, were not performed during the acute hospitalization, and outpatient cardiac MRI had not yet been completed at the time of this report. Long-term follow-up data beyond the early post-hospital period remain limited, and the single-center experience restricts generalizability. In addition, treatment decisions, including empiric rituximab dosing and the combination of abatacept with ruxolitinib, were guided by emerging literature and evolving expert practice rather than a validated protocol specific to MMM overlap syndrome. Additionally, an outpatient follow-up MRI of the brain to characterize the incidentally identified sphenoid wing lesion had not yet been completed at the time of this report.

## Conclusions

Our case highlights the need for escalation to additional lines of immunosuppression in patients with severe myocarditis associated with MMM overlap syndrome. It also underscores the importance of actively screening for the other components of the syndrome whenever one of the three entities is identified. In our case, myositis and myasthenia gravis were apparent on initial presentation, and elevated troponin subsequently led to the diagnosis of concurrent myocarditis. Further research is needed to clarify any association between malignancy type and the risk of developing this syndrome, and to identify risk factors and biomarkers that may help identify high-risk patients. Standardization of treatment protocols and greater clinician awareness of this emerging entity are both essential for early identification and intervention. A multidisciplinary team approach is critical for managing this often-fatal condition.
